# Effect of oxytocin on the survival of random skin flaps

**DOI:** 10.18632/oncotarget.21696

**Published:** 2017-10-09

**Authors:** Peng-Fu Xu, Miao-Jie Fang, Yu-Zhi Jin, Le-Sha Wang, Ding-Sheng Lin

**Affiliations:** ^1^ Department of Hand Surgery, The Second Affiliated Hospital and Yuying Children's Hospital of Wenzhou Medical University, Wenzhou, China

**Keywords:** random skin flap, oxytocin, VEGF, ischemia-reperfusion, inflammation

## Abstract

Random flap transplantation is widely used to repair and rebuild skin soft tissue. However, such flaps exhibit poor survival. Plastic surgeons seek to improve flap survival. We explored whether oxytocin improved skin flap survival. Overlength random skin flaps (9 × 3 cm) were established on backs of 80 healthy male SD rats randomly divided into two groups. One group was injected daily with oxytocin (1 mg/kg; test group) and the other with normal saline (control group). On postoperative day 2, malondialdehyde (MDA) and superoxide dismutase (SOD) levels were measured. On postoperative day 7, the flap survival area was measured using transparent graph paper. Microvessel numbers were evaluated histologically by hematoxylin and eosin staining. VEGF expression was assessed immunohistochemically. Angiogenesis was evaluated via lead oxide–gelatin angiography and blood flow via laser Doppler flowmetry. In the test group compared with the control group, the flap survival rate and SOD activity were increased markedly, the MDA level was decreased, and according to hematoxylin and eosin staining, inflammation was significantly attenuated. In addition, the test group exhibited higher levels of VEGF and skin flap angiogenesis. Oxytocin improved flap survival rate by increasing microcirculation and angiogenesis and attenuating ischemia–reperfusion injury.

## INTRODUCTION

Random skin flaps are widely used in the repair of the reconstruction of several tissue defects and local tissue loss attributable to trauma, congenital disorders, cancer, excisions, and other causes [1]. It is challenging that postoperative flap necrosis is a common complication. Although flap design and the surgical techniques used have improved, the flap length-to-width ratio usually cannot exceed 1.5–2, limiting the clinical applications [2]. However, the ratio can be as high as 3 in local blood-rich areas such as the face. If the length-to-width ratio is not thus constrained, a certain proportion of the flap is prone to ischemic necrosis, and random skin flap transplantation is thus associated with a 10–20% failure rate [3]. Complete or incomplete ischemia of the skin flap is a widespread postsurgical problem [4]. As we all know, ischemia is associated with inadequate blood flow and disturbed venous drainage [5–7]. Therefore, the principal strategies promoting skin flap survival are inhibition of ischemia–reperfusion injury, acceleration of angiogenesis, and alleviation of tissue edema [2, 8, 9].

Oxytocin (OT), a posterior pituitary hormone, affects many biological processes including uterine contraction, learning, memory, feeding, mental behavior, reproductive and sexual behavior, pain, and body temperature. OT even has the effect on the metabolism regulation and tumor like prostate cancer promotion effects, which may be a biomarker for prostate cancer in the future [10]. OT is an important circulatory regulator, controlling blood pressure and the levels of blood electrolytes. Extremely, OT may act via the release of nitric oxide or atrial natriuretic peptide or by effects on the adrenergic receptor [11, 12]. The effects of OT on hemodynamics are dose-dependent. Rapid administration of large OT doses can cause blood pressure to decrease [13–15]. In 1988, M Petersson et al. reported that OT increased the survival of musculocutaneous flaps. They found that the plasma levels of insulin-like growth factors(IGF-1) and nerve growth factor(NGF) were significantly increased in OT-treated rats. Thus, OT may promote the release of multiple growth factors to protect musculocutaneous flaps [16]. There was little research about mechanism in this article. Musculocutaneous flap compared with random skin flap, having pretty rich muscles and blood vessels [17], causes so many factors influencing the detection about effect of OT on flap. Nevertheless, random skin flap model is classic, which can detect the role of drugs on flaps direcly [18], and it is not known whether OT promotes skin flap survival. Thus, we investigated the effect and potential mechanism of OT on the survival of random skin flap more deeply.

## RESULTS

### General

**Figure 1:**
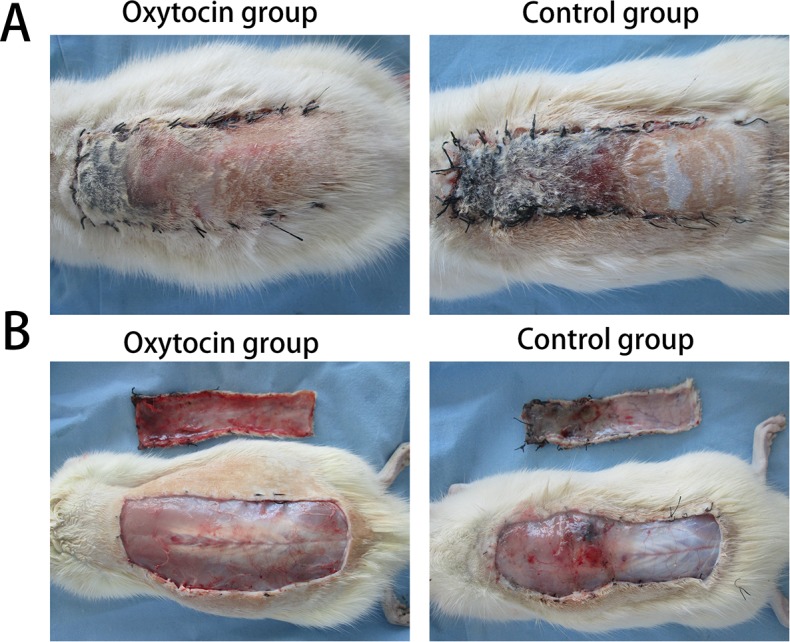
Skin flap survival and edema on postoperative day 7 **(A)** In the oxytocin group, region II was pink and elastic, without a callus, and region III was black with dry crusts. **(B)** In the control group, both regions II and III showed poor elasticity, black color, and dry crusts. (B) The photographs revealed more pronounced edema in the control than in the oxytocin group.

All rats survived the protocol, and no infections or deaths were observed. The flaps of both groups exhibited varying extents of swelling with rather pale pedicles. The distal areas (region III) were dark purple without obvious necrosis. On postoperative day 5, the edema of the region I of the two groups gradually subsided. Local focal and small-sheet necrosis (reddish-brown in color with congestion) were evident in regions II and III. On day 7, all region I of both groups was viable, as were parts of region II. Both regions I and II bore fine hair. However, all of region III was necrotic (Figure 1A). In addition, the granulation tissue under the surviving flaps was much thinner in the OT group than in the control group. More bleeding scattered in subcutaneous was evident in the OT group. The control group flaps exhibited more obvious edema than did the OT group flaps (Figure 1B).

### Flap survival

**Figure 2:**
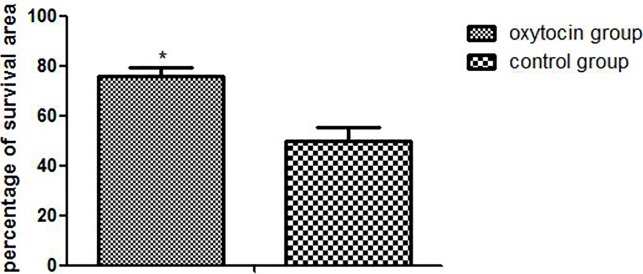
The flap survival rates in the two groups (^*^p < 0.01 vs. the control group)

The flap survival rate in the OT group was much higher than that in the control group (75.82 ± 3.74% vs. 49.81 ± 5.35%, respectively; Figure 2) (p < 0.01).

### Histopathological changes

**Figure 3:**
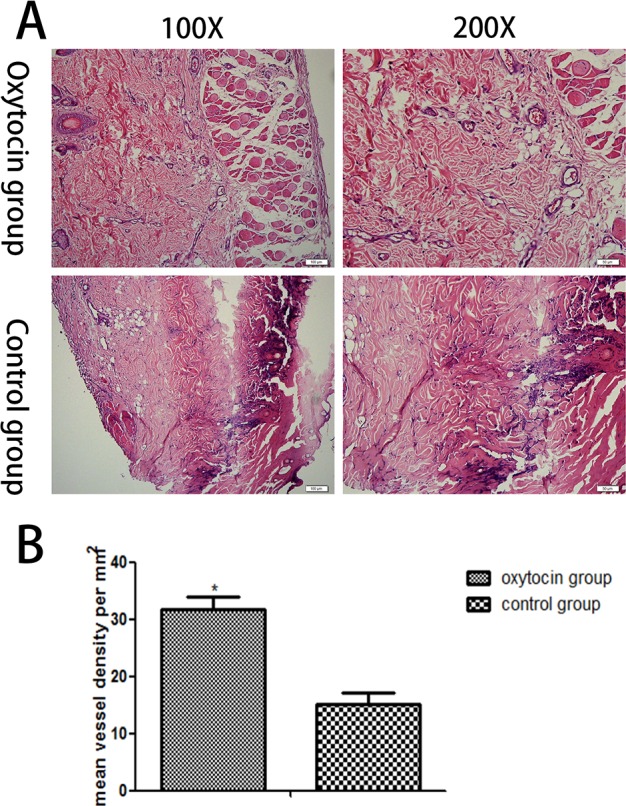
Histopathological changes in the skin flaps of the two groups **(A)** Histopathological changes in flap region II (original magnifications: ×100 and ×200). **(B)** The mean malondialdehyde levels in region II of the two groups (^*^p < 0.01 vs. the control group).

Flaps were stained with hematoxylin and eosin and observed under a light microscope. In flap region II, the control group exhibited less fibroblastic proliferation, fewer new blood vessels, thicker neutrophil infiltration, and more edema, compared with the OT group (Figure 3A). In addition, region III of the control group exhibited severe necrosis. The microvascular density of region II was significantly higher in the OT than the control group (31.83 ± 2.03/mm2 vs. 15.23 ± 1.86)/mm2, respectively; p < 0.01; Figure 3B).

### Superoxide dismutase (SOD) activity and malondialdehyde (MDA) content

**Figure 4:**
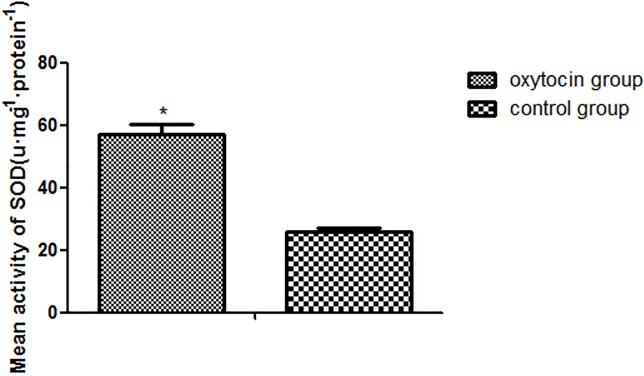
SOD expression levels in the two groups (^*^p < 0.01 vs. the control group) All values are means ± standard deviations (SDs). n = 6 per group.

**Figure 5:**
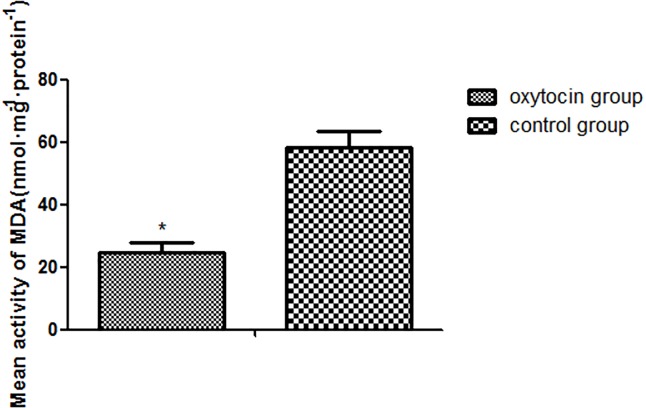
Mean MDA expression levels in the two groups (^*^p < 0.01 vs. control group) All values are expressed as means ± standard deviations (SDs). n = 6 per group.

The mean SOD activity was significantly higher in the OT group than in the control group (25.73±1.43 units/mg protein vs. 56.91±3.32 units/mg protein, respectively; p < 0.01; Figure 4). The mean MDA level of the OT group was 24.70±6.30 nmol·mg/protein, significantly lower than that in the control group (58.41±5.00 nmol·mg/protein (p < 0.01; Figure 5).

### VEGF expression

**Figure 6:**
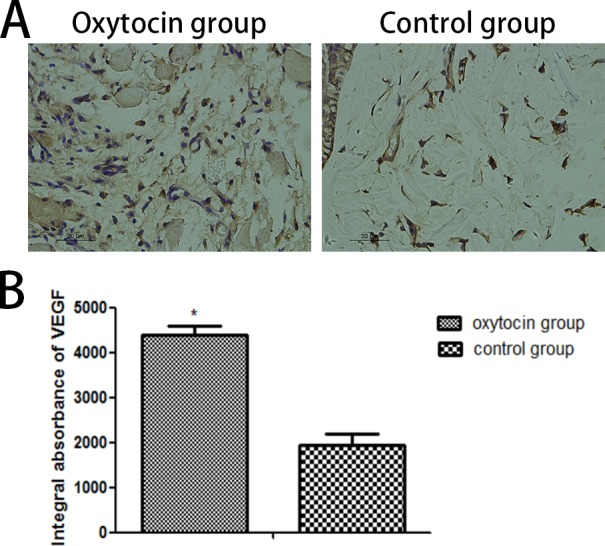
VEGF levels in flap region II of the two groups **(A)** Immunohistochemical light microscopic images (original magnification: ×400). **(B)** Integral absorbances (IAs) of the VEGF levels (^*^p < 0.01 vs. control). All values are expressed as means ± standard deviations (SDs). n = 6 per group.

Immunohistochemical VEGF staining (Figure 6A) revealed that VEGF expression was much higher in the OT than in the control group (4,381.50 ± 219.75 IA vs. 1,948.50 ± 233.64 IA, respectively; p < 0.01; Figure 6B).

### Microangiographic vascularization

**Figure 7:**
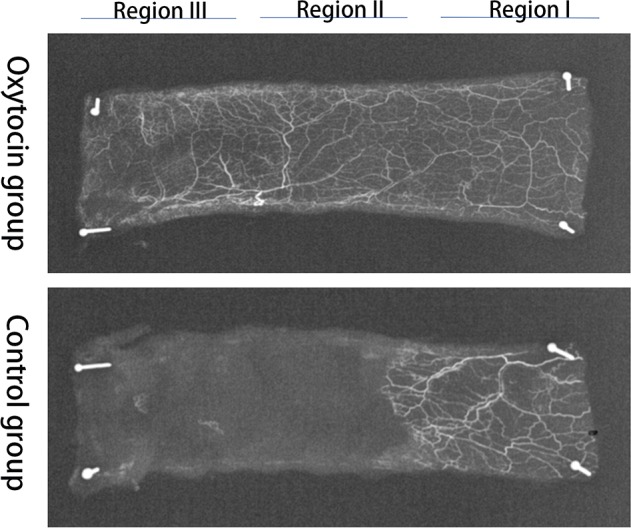
X-ray microangiographic images of the flaps in the two groups obtained 7 days postoperatively

Angiography revealed no significant differences in the vascular images or flap survival rates between the two groups. Flap microvascular development, neovascularization, and imaging changes were all clearly higher in the OT than in the control group on postoperative day 7 (Figure 7).

### Flap blood perfusion

**Figure 8:**
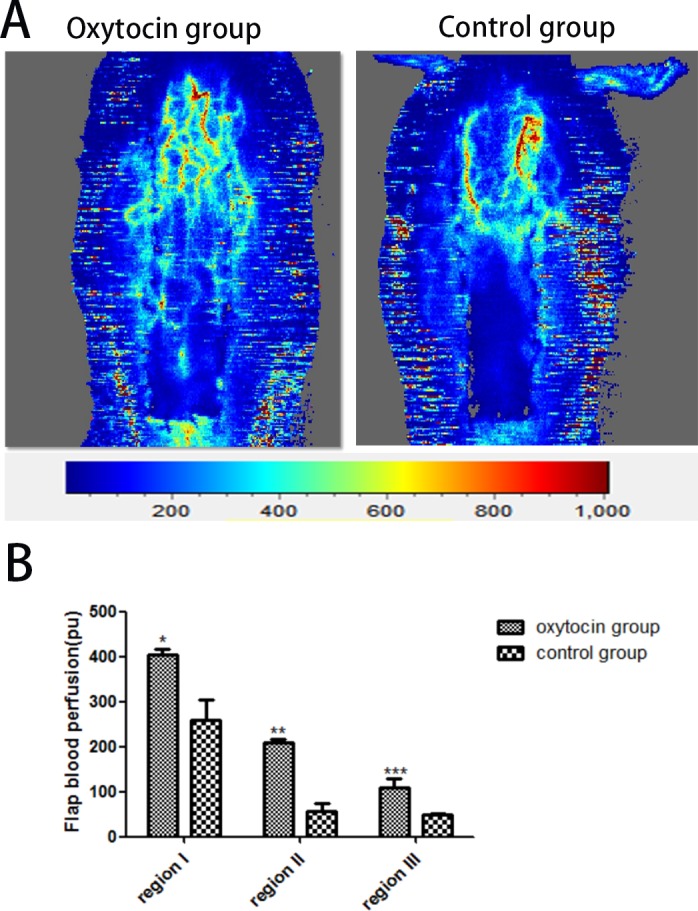
Flap blood flow measurements **(A)** Representative digital images of blood perfusion in the two groups. **(B)** The mean blood perfusion levels in regions I–III of the two groups. ^*^p < 0.01 vs. the control group). All values are expressed as means ± standard deviation (SDs). n = 6 per group.

The Doppler flowmeter images are shown in Figure 8A. The blood perfusions in the control group in regions I (259.82 ± 45.04 pu), II (56.77 ± 18.28 pu), and III (48.00 ± 4.26 pu) were much lower than those in the OT group (403.82 ± 13.21 pu, 209.43 ±6.93 pu, and 109.95 ±19.59 pu, respectively). The differences were statistically significant (all p < 0.01; Figure 8B).

## DISCUSSION

Random skin flap transplantation has been used to repair and rebuild skin soft tissue. Flap transfer is technically simple, and flap color and texture can be matched to those of the recipient site [19, 20]. However, distal flap necrosis remains a major challenge [21, 22]. It was demonstrated that OT injection significantly increases the plasma levels of IGF-1 and NGF and improves musculocutaneous flap survival [16]. However, musculocutaneous flap is a compound tissue flap utilizing a piece of muscle (or part of muscle) from body and is along with its shallow subcutaneous tissue, which is transfered for repair of large wound defects or reconstruction of muscle function and whose pedicle is the vascularities entering the muscle [23–25]. With rich muscles and vascularities, musculocutaneous flap causes so many factors influencing the detection about effects of drugs on flaps. Compared with musculocutaneous flap, random skin flap is the flap that doesn't contain shaft blood vessels or well-known vessels [18], so we choosed random skin flap model for more intuitive study about machanism of OT on flaps.

In the present study, we found that OT improved the flap survival rate by attenuating ischemia–reperfusion injury and tissue inflammation and increasing angiogenesis. Flap necrosis is caused by ischemia–reperfusion injury, an inadequate blood supply, and venous congestion [26, 27].

Ischemia–reperfusion injury, a complex process featuring inflammatory oxidation [28], is one of the main causes of flap necrosis caused by increased production of oxygen free radicals. SOD is an antioxidant enzyme that plays an important role in the oxidation/anti-oxidation balance. SOD removes the superoxide anion free radical, thus attenuating ischemia–reperfusion injury and protecting cells [29]. Therefore, the SOD level indirectly reflects the ability to scavenge oxygen free radicals. We found that the SOD level in the OT group was significantly higher than that in the control group on postoperative day 7, indicating that OT increased the SOD content and antioxidant capacity of flap tissue.

MDA is the most common product of lipid peroxidation when oxygen-based free radicals attack the cell membrane [30]. Thus, MDA levels indirectly reflect the extent of free radical attack. The higher the MDA level, the greater the intensity of attack and thus more severe oxidative damage [31]. A lower MDA level reflects less flap ischemia–reperfusion injury, improving flap survival. We found that the MDA level was lower in the OT flaps than in the control flaps on day 7 after grafting, indicating that OT reduced the flap MDA level by inhibiting lipid peroxidation, thus reducing ischemia–reperfusion injury.

VEGF is a highly specific mitogen that increases the vascular permeability of endothelial cells. Its principal receptors are VEGFR-I and VEGFR-2. VEGF binding to the receptors induces Ca^2 +^ inflow into the vascular endothelium rapidly, triggering the release of factor VIII. VEGF also increases the intracellular IP3(inositol 1, 4, 5-triphosphate) concentration by activating phosphoinositol-specific phospholipase C; IP3 enhances microvascular permeability and promotes endothelial cell division, migration, and vascular constriction [32, 33]. The flap VEGF level greatly influences flap vessel growth [34]. VEGF improves the survival rate of full-thickness flaps by reducing flap necrosis. In the present study, the level of VEGF expression and the microvessel density (a measure of neovascularization) were clearly greater in flaps of the OT group than of the control group. Moreover, angiography and the blood perfusion data showed more microvessels in OT flaps than control flaps, indicating that OT may protect the flaps via VEGF-mediated angiogenesis. However, further study is required.

Recent studies have shown that subcutaneous OT did not bind to any receptor in the flaps [35]. However, exogenous OT can cross the blood–brain barrier [36]. Thus, subcutaneous OT may enter the brain, trigger the brain OT receptor, and thus protect against skin flap injury caused by ischemia–reperfusion.

In conclusion, we demonstrated that OT injection improved the flap survival rate in rats by enhancing microcirculation, increasing angiogenesis, and attenuating ischemia–reperfusion injury.

## MATERIALS AND METHODS

### Animals

A totla of eighty healthy male Sprague–Dawley(SD) rats weighting between 250 and 280g were purchased from Wenzhou Medical University (license no. SCXK[ZJ]2005 - 0019) with the approval and permission from the Guide for the Care and Use of Laboratory Animals of China National Institutes of Health and the Animal Care and Use Committee of Wenzhou Medical University (wydw2012 - 0079). Rats used in this study were divided randomly into two equal sized groups: the control group and the oxytocin group, with 40 rats each group.

### Flap animal model

**Figure 9:**
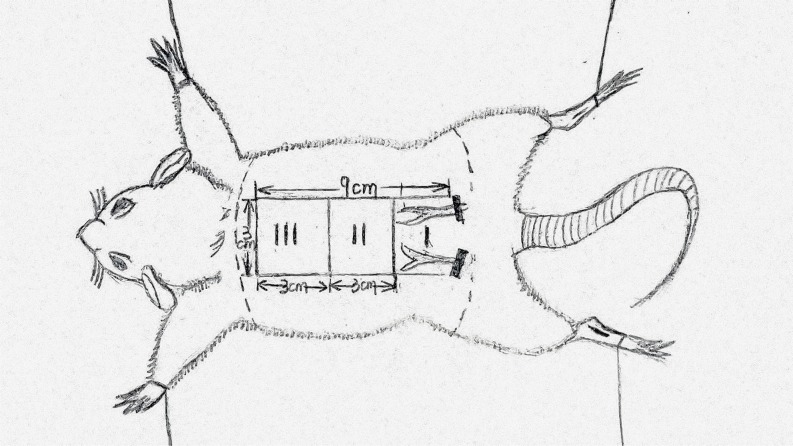
The flap(3cm×9cm) area was divided into three equal sized zones and marked as proximal(region I), intermediate (region II) and distal (region III)

All rats were anesthetized by intraperitoneal injection of 2% (w/v) pentobarbital sodium (40mg/Kg, Solarbio Science & Technology, Beijing, China). A McFarlane flap model whose procedure was modified based on the method reported by Kelly CP was established on rat dorsum (the same position in all rats) [18]. Laboratory rats were ventilated and fixed on a thermostatic operating table. Barium sulfide: talc: flour = 1: 1: 1 with water stirred into a paste depressant was applied to the rat dorsum. After the removal of the hair and conventional disinfection, rat dorsal as the longitudinal axis of the flap and connection of rat lliac crests as the pedical, the deep fascia shallow and flesh deep dissection between the anatomy were set off to establish a random skin flap (9cm×3cm). Immediately, flaps were sutured to the donor bed using a wedged-on cutting needle and continuous 4-0 silk sutures. The area around incision was disinfected by the poly-cup copper iodine and smeared with chlortetracycline ointment. The surgical procedures were implemented strictly by one experimental staff under aseptic conditions. For the interest of analysis, the flap area was divided into three equal sized zones (Figure 9) and marked as proximal (region I), intermediate (region II) and distal (region III).

### Experimental protocol

**Figure 10:**
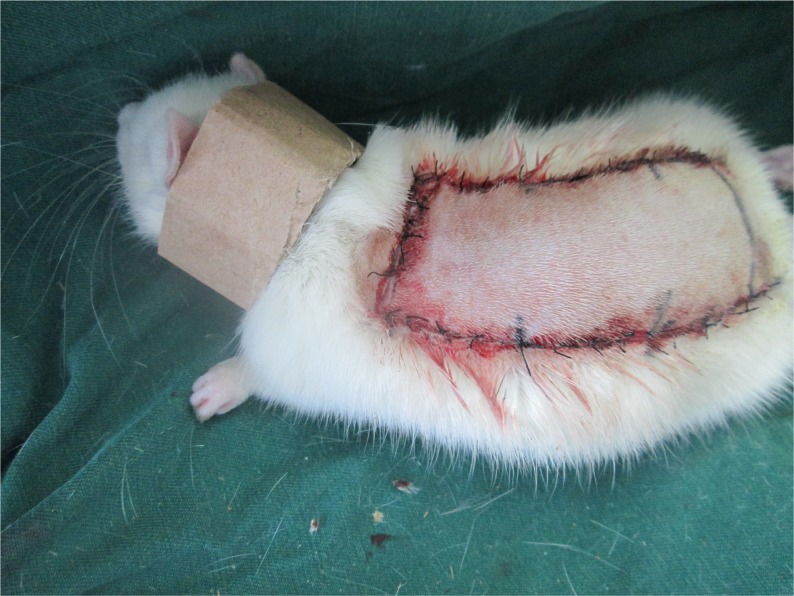
Each rat was equiped with a neck collar to prevent injury caused by self-mutilation

OT (1 mg/kg/d, subcutaneous injection) was injected into the thigh muscles of the rats in the oxytocin group(n=40) for seven days, while the control group (n =40) received equal volumes of saline solution. And rats were individually housed in comfortable cage at 22-25°C with adequate food and drink. No rats died during the procedure. Each rat was equiped with a neck collar (Figure 10) to prevent injury caused by self-mutilation [37]. All rats removed from the study were euthanized with an overdose of pentobarbital sodium for minimizeing suffering.

### Macroscopic evaluation and assessment of surviving areas

After injection, each flap survival area was observed visually during the 7 days, including flap appearance, color, texture and hair condition macroscopic changes developing. Rats dorsum in each group were covered with clear paper in anesthesia to accurately measure the survival or necrotic area of the flap and cut into two parts: survival and necrosis. According to the following formula to calculate the flap survival area ratio:

flap survival area ratio = (survival area cellophane quality / total flap area cellophane quality) × 100%.

### Histologic examination

On postoperative day 7, laboratory rats were sacrificed by an overdose of pentobarbital sodium and pieces of random skin flap(1cm×1 cm) were collected from three equal sections marked as regions I to III and biopsied for histology. Samples were fixed in 10% paraformaldehyde for 24 hours and routine procedures were performed. All samples were embedded in paraffin, cut into 4 um slices and strained with hematoxylin and eosin (H&E). The granulation tissue thickness, tissue edema, necrosis, inflammatory cell infiltration were observed under a light microscope (×100 magnification). The microvessel density detection (MVD) can also be carried out. The method is as follows: 1 slice per group, each slice in the central area and the surrounding area were randomly selected five high times (×200 magnification) field of view, the blood vessels count to take the average. Each high magnification field of view was 0.305 mm^2^, calculating the number of microvessel per unit area (/ mm^2^) as an indicator of microvessel density.

### Immunohistochemistry for VEGF

The expression of VEGF was assessed immunohistochemically. The remaining paraffin sections from H&E were subjected to Elivison two-step staining. Firstly, each sample was added with normal goat serum blocking solution, allowed to stand for 20 min at room temperature, and then added with 50ul mouse anti-rat VEGF antibody (primary antibody, working concentration 1: 100). Secondly, we let slices stay at 4°C overnight. Following this, samples were rewarmed at 37°C for 45 min and rinsed with phosphate buffer solution(PBS). Fourthly, dropping 50ul goat anti-mouse secondary antibody (working concentration 1:50), the slices were incubated at 37°C for 1 hour and washed with PBS. Fifthly, for the sake of color development, we incubated the slices in 3,3-diaminobenzidinetetrahydrochloride (DAB) solution for 5 ∼ 10 min. The BX51 optical microscope (Olympus, Japan) was used to find the positive expression region of VEGF at a low magnification. Next, the DP2-TWAIN image acquisition system (Olympus, Japan) was selected by observing each slice with high magnification. Fianlly, the integral absorbance(IA) values was read using Image-Plo Plus v 6.0 software (US Media Cybernctics) to represent VEGF expression levels.

### Superoxide dismutase (SOD) activity and malondialdehyde (MDA) content

Oxidative stress status of the flaps was accessed via Superoxide dismutase (SOD) and malondialdehyde (MDA) test kits (Nanjing Jiancheng Biology Institution, Nanjing, China). At the 2th day after operation, 30 whole layers (0.5 cm×0.5 cm) were obtained from the middle part between region II and III. Then the cinnamon layer was removed. The mass was weighed and homogenized. Finally, the volume fraction of 10% tissue was prepared in an ice bath. We determined to detected SOD activity by using the xanthine oxidase method and estimate MDA content via reacting with thiobarbituric acid at 90–100°C [38].

### Gelatin-lead oxide angiography

On day 7, we picked out 7 rats from each group. 1.5 mL 1% heparin saline was injected into the carotid artery of rats. Then, the gelatin lead perfusion fluid (a mixture of lead oxide, gelatin, and water, 150ml / kg) was injected slowly into the carotid artery. We didn't stop injectting until the rat sclera and extremity of the limb became the color of the contrast agent. After perfusion, rats were frozen for 24 hours and the gelatin was agglutinated. Finally, the dorsal skin flap and the surrounding skin were dissected and flap was radiographed (54 kVp, 40 mA, and 100 second-exposure) with a X-ray machine.

### Measurement of flap blood flow

We obtained laser Doppler flowmeter measurement for all rats. On the 7th day after operation, the relative blood flow of the flap(region I, region II, region III) was measured with the Laserflo BPM2(Vasamedics, St. Paul, MN, USA)(Normal blood flow was adjusted to 100%), which measured Pu value (blood perfusion unit). Each area was measured 6 times and averaged, representing the part of the average blood flow value.

### Statistical analysis

In this study, all values was expressed as the mean ± standard deviations (SD). Student t-test was applied for comparisons between 2 means. The Mann–Whitney test was used to compare percentage survivals, histological and immunohistochemical results. All statistical analyses were conducted using SPSS software (ver.19.0; SPSS Inc., Chicago, IL). Statistical significance was set at p values < 0.05.

## Abbreviations

OT: oxytocin
H&E: hematoxylin and eosin
MDA: malondialdehyde
SOD: superoxide dismutase
VEGF: vascular endothelial growth factor
H&E: hematoxylin and eosin
IA: integral absorbance
IGF-1: insulin-like growth factors
NGF: nerve growth factor
SD: standard deviations
PBS: phosphate buffer solution
DAB: 3,3-diaminobenzidinetetrahydrochloride
